# Supplementary material for: Changes in the bacterial community colonizing extracted and non-extracted tannin-rich plants in the rumen of dromedary camels
Source: PLoS One. 2023 Mar 10;18(3):e0282889. doi: 10.1371/journal.pone.0282889 (PMC10004507; doi:10.1371/journal.pone.0282889)
Supplement: S1 Table — (DOCX) [file pone.0282889.s001.docx]

Supplementary Table S1: The relative abundance (%) of rare bacterial phyla and bacterial families and genera colonized to extracted and non-extracted atriplex, acacia, and leucaena at 6 and 12 hours incubations.

| Item | **Atriplex** | | | | **Acacia** | | | | **Leucaena** | | | |
| --- | --- | --- | --- | --- | --- | --- | --- | --- | --- | --- | --- | --- |
|  | **AN (Non-Extracted)** | | **AE(Extracted)** | | **CN(Non-Extracted)** | | **CE(Extracted)** | | **LN(Non-Extracted)** | | **LE(Extracted)** | |
|  | **6h** | **12h** | **6h** | **12h** | **6h** | **12h** | **6h** | **12h** | **6h** | **12h** | **6h** | **12h** |
| **Bacterial phyla that were not observed in all samples** | | | | | | | | | | | | |
| Actinobacteria | 0.8 |  |  |  |  |  | 0.08 |  |  |  |  |  |
| Chloroflexi |  | 0.09 |  |  |  |  | 0.08 |  |  |  |  |  |
| Cyanobacteria | 0.1 | 0.085 | 0.1 |  | 0.6 | 0.9 | 0.2 |  | 1.6 | 0.7 | 1.2 | 1.3 |
| Fibrobacteria |  |  | 0.03 | 0.1 |  |  | 0.1 | 0.15 |  | 0.5 | 0.08 | 0.29 |
| Fusobacteria | 0.04 |  |  |  |  |  |  |  | 0.06 |  | 0.05 |  |
| Kiritimatiellaeota | 0.3 | 0.18 | 0.13 |  | 0.1 |  | 0.15 |  | 0.12 |  |  | 0.15 |
| Lentisphaerae |  |  |  |  |  |  | 0.07 |  |  |  |  |  |
| Patescibacteria | 0.07 |  |  |  |  |  |  |  |  |  |  |  |
| Synergistetes | 0.14 | 0.15 | 0.13 | 0.14 | 0.22 | 0.16 | 0.2 | 0.26 | 0.2 | 0.1 | 0.3 | - |
| Verrucomicrobia | 0.14 |  | 0.02 |  | 0.23 | 0.18 | 0.06 | 0.07 |  |  | 0.13 |  |
| **Bacterial families and genera** | | | | | | | | | | | | |
| Prevotellaceae**, F** | 44.3 | 44.8 | 64.1 | 44.3 | 45.3 | 42.1 | 39.2 | 40.06 | 52.5 | 53. 3 | 54.6 | 64.2 |
| Prevotella_1,**G** | 35.9 | 31.3 | 56.4 | 34.2 | 31.5 | 31.5 | 25.9 | 27.2 | 38.3 | 40 | 41 | 54 |
| Prevotella_7, **G** | 1.2 | 5.2 | 1.4 | 1.2 | 2.3 | 2.4 | 0.5 | 0.8 | 0.8 | 1.4 | 0.4 | 2.1 |
| Ga6A1_group, **G** | 0.05 | 0.1 | 0.37 | 0.24 | 0.16 | 0.4 | 0.16 | 0 | 0 | 0.6 | 0.6 | 0.34 |
| YAB2003_group, **G** | 0.4 | 0.9 | 0 | 0 | 0.6 | 1 | 0.4 | 0 | 0 | 0.5 | 0.17 | 0.4 |
| Alloprevotella, **G** | 0.06 | 0 | 0 | 0.12 | 0.35 | 0.2 | 0 |  | 0.24 | 0 | 0 | 0 |
| UCG-004, **G** | 0.1 | 0 | 0 | 0 | 0 | 0.2 | 0.1 | 0 | 0 | 0.08 | 0 | 0.05 |
| P: Bacteroidetes; C: Bacteroidia; O: Bacteroidales; F: Rikenellaceae | | | | | | | | | | | | |
| Rikenellaceae, F | 8.3 | 7.6 | 6.7 | 6.5 | 8.5 | 8 | 6.3 | 7 | 6 | 7.9 | 4.7 | 6.5 |
| RC9_gut_group,**G** | 8 | 7.4 | 6.4 | 6.3 | 8.3 | 8 | 6 | 6.9 | 5.9 | 7.7 | 4.5 | 6.3 |
| U29-B03,**G** | 0.25 | 0.14 | 0.3 | 0.15 | 0.2 | 0.1 | 0.25 | 0.25 | 0.14 | 0.17 | 0.26 | 0.13 |
| NA ( Non- classified),**G** | 6.6 | 7.6 | 7.6 | 17 | 6.6 | 10.7 | 21.1 | 12.7 | 13.2 | 9.2 | 12.8 | 7.35 |
| P: Bacteroidetes; C: Bacteroidia; O: Bacteroidales | | | | | | | | | | | | |
| **F:** F082; NA, **G** | 3.8 | 5.1 | 5.6 | 5.7 | 7.8 | 5.3 | 6.2 | 6.2 | 5.8 | 7.3 | 4.7 | 5.8 |
| **F:** p-251-o5; NA, **G** | 2 | 2 | 1.4 | 12.8 | 13.9 | 16.1 | 9.2 | 21.15 | 6.2 | 7.4 | 8.9 | 3 |
| **F**: NA; NA, **G** | 0.6 | 0.56 | 0.4 | 0.5 | 0.5 | 0.45 | 0.8 | 0.85 | 0.5 | 0.3 | 0.5 | 0.25 |
| **F**: PeH15; NA, **G** | 0.32 | 0.2 | 0.23 | 0.25 | 0.6 | 0.26 | 0.37 | 0.4 | 0.26 | 0.35 | 0.25 | 0.37 |
| **F**: S11_gut_group; NA, **G** | 0 | 0.14 | 0.12 | 0.25 | 0.4 | 0.27 | 0.29 | 0.25 | 0.11 | 0.28 | 0.10 | 0.26 |
| **F:** UCG-001; NA, **G** | 0.07 | 0 | 0.22 | 0.19 | 0.92 | 0.15 | 0.24 | 0.43 | 0.26 | 0.34 | 0.33 | 0.14 |
| P: Cyanobacteria ; C: Oxyphotobacteria ; O: Chloroplast | | | | | | | | | | | | |
| NA; **F** NA**,G** | 0.08 | 0 | 0.08 | 0 | 0.56 | 0.8 | 0.17 | 0 | 1.56 | 0.67 | 1.1 | 1.26 |
|  | | | | | | | | | | | | |
| **F:** Lachnospiraceae | 10.9 | 17.8 | 2.80 | 3.2 | 2.7 | 3.06 | 3.3 | 2.3 | 4.3 | 3.7 | 3.8 | 3.3 |
| NA, **G** | 2.1 | 1.3 | 0.65 | 1.08 | 0.6 | 0.37 | 0.8 | 1.04 | 08 | 0.8 | 0.9 | 0.9 |
| Oribacterium**, G** | 3.06 | 3.67 | 0.11 |  | 0 |  | 0 |  | 0.17 |  | 0.19 |  |
| Shuttleworthia**, G** | 0.66 | 1.01 |  | 0.27 |  |  |  |  |  | 0.5 |  | 0.53 |
| Acetitomaculum, **G** | 0.22 | 0.15 | 0.22 |  | 0 |  | 0.6 |  | 0.1 |  | 0.36 |  |
| Butyrivibrio_2, **G** | 1.1 | 0.9 | 0.2 | 0.31 | 0.6 | 1.1 | 0.55 | 0.46 | 0.5 | 0.7 | 0.3 | 0.23 |
| AC2044_group, **G** | 0.52 | 0 | 0 | 0 | 0.2 | 0.15 | 0.009 | 0.1 | 0.3 | 0.19 | 0 | 0 |
| XPB1014_group, **G** | 1.6 | 0.9 | 1.07 | 0.75 | 0.25 | 0.27 | 0.8 | 0.32 | 1.16 | 0.77 | 1.24 | 0.9 |
| NK3A20_group, **G** | 0.34 | 0.31 | 0.12 | 0.25 | 0.31 | 0.74 | 0.20 | 0 | 0.5 | 0.6 | 0.32 | 0.4 |
| probable_genus_10, **G** | 0.3 | 0.1 |  | 0.18 |  |  |  |  | 0.17 | 0.1 |  |  |
| UCG-008, **G** | 0.15 |  | 0.075 | 0.075 |  |  |  |  |  | 0.1 |  |  |
| Moryella, **G** | 0.16 |  | 0.16 |  | 0.22 |  |  |  | 0.5 |  | 0.4 |  |
| Lachnoclostridium_1 | 0.12 | 0.24 |  |  |  |  |  |  |  |  |  |  |
| Lachnoclostridium_10, **G** | 0.06 |  | 0.08 |  |  |  |  |  | 0.07 |  |  |  |
| ND3007_group, **G** | 0.13 |  |  |  |  |  |  |  |  |  |  | 0.04 |
| FCS020_group, **G** | 0.11 |  |  |  |  |  |  |  |  |  |  |  |
| UCG-006, **G** | 0.07 |  |  |  |  |  |  |  |  |  |  |  |
| Marvinbryantia, **G** | 0.07 |  |  |  |  |  |  |  |  |  |  |  |
| Lachnospiraceae_NK4A136_group, **G** | 0.1 |  |  |  |  |  |  |  |  |  |  |  |
| **P**: Firmicutes ; **C:** Clostridia ; **O**: Clostridiales : F: Ruminococcaceae | | | | | | | | | | | | |
| **F**: Ruminococcaceae | 7.3 | 3 | 4.7 | 3.9 | 3.8 | 3.1 | 6.6 | 4.2 | 3.8 | 3.6 | 3.7 | 3.4 |
| UCG-014, **G** | 1.45 | 0.63 | 1.02 | 1.07 | 0.6 | 0.46 | 2.27 | 1.3 | 0.52 | 0.75 | 0.65 | 0.6 |
| Saccharofermentans, **G** | 0.84 | 0.57 | 0.65 | 0.5 | 0.25 | 0.16 | 0.35 | 0.14 | 0.5 | 0.26 | 0.35 | 0.5 |
| UCG-004, **G** | 0.25 | 0.2 | 0.35 | 0.5 | 0.5 | 0.1 | 0.39 | 0.46 | 0.39 | 0.37 | 0.59 | 0.15 |
| NK4A214_group, **G** | 1.78 | 0 | 1.38 | 0.76 | 1.5 | 0.45 | 1.9 | 0.69 | 1.26 | 0.6 | 1.2 | 1.17 |
| Ruminococcus_1, **G** | 0.44 | 0.2 | 0.23 |  | 0.27 | 0.2 |  |  |  | 0.3 |  | 0.32 |
| Ruminiclostridium_6, **G** | 0.25 | 0.2 | 0.16 | 0.22 | 0 | 0.18 | 0.25 | 0 | 0.44 | 0.21^a^ | 0.5 | 0.3 |
| Ruminococcus_2, **G** | 0.38 | 0.3 | 0.2 | 0.5 | 0.25 | 0.14 | 0.43 | 0.6 | 0.05 |  | 0.23 | 0.23 |
| NA, **G** | 1 | 0.1 | 0.27 | 0.2 | 0.43 | 0.4 | 0.35 | 0.3 | 0.23 | 0.2 | 0.26 | 0.3 |
| UCG-005, **G** | 0.26 | 0.16 | 0.16 | 0 | 0.27 | 0.08 | 0 | 0 | 0.21 | 0.12 | 0 | 0 |
| Papillibacter, **G** | 0.13 |  |  |  |  | 0.3 |  | 0.23 |  |  |  |  |
| UCG-010, **G** | 0.4 |  | 0.25 |  | 0.3 | 0.13 | 0.24 | 0.2 | 0.16 | 0.2 | 0.1 |  |
| Caproiciproducens, **G** | 0 |  | 0.13 |  | 0 |  | 0.12 |  | 0.1 |  |  |  |
| UCG-002, **G** | 0.08 |  |  |  |  |  |  |  |  |  |  |  |
| **P:** Firmicutes ; **C:** Negativicutes; **O**: Selenomonadales **: F**: Veillonellaceae | | | | | | | | | | | | |
| Veillonellaceae, **F** | 5.4 | 5 | 0.93 | 0.6 | 4.7 | 5.8 | 0.66 | 0.48 | 3.1 | 1.09 | 0.9 | 1.0 |
| Selenomonas_1, **G** | 4.4 | 4.6 | 0 | 0 | 3.3 | 4.1 | 0 | 0.35 | 1.98 | 0.87 | 0.48 | 0.46 |
| Anaerovibrio, **G** | 0.43 | 0.33 | 0.15 | 0.26 | 0 | 0 | 0 | 0 | 0 | 0 | 0 | 0 |
| Schwartzia, **G** | 0.66 | 1 | 0.1 |  |  |  |  |  |  |  |  | 0.13 |
| Selenomonas, **G** | 0.59 | 0.49 | 0 | 0 | 0.66 | 0.9 | 0 | 0 | 0.38 | 0.34 | 0 | 0 |
| NA, **G** | 0.43 | 0.2 | 0.18 | 0 | 0.77 | 1.4 | 0.2 | 0 | 0.4 | 0.16 | 0 | 0.2 |
| UCG-001, **G** | 0.44 | 0.28 | 0.44 | 0.36 | 0.14 | 0 | 0.16 | 0.11 | 0.23 | 0.17 | 0.26 | 0.24 |
| **P:** Firmicutes **; C:** Clostridia ; **O**: Clostridiales **: F:** Family_XIII | | | | | | | | | | | | |
| Family_XIII, **F** | 0.71 | 0.3 | 0.33 | 0.36 | 0.27 | 0.14 | 0.37 | 0.3 | 0.34 | 0.3 | 0.27 | 0.1 |
| Anaerovorax, **G** | 0.45 | 0.27 | 0.23 | 0.21 | 0.24 | 0.11 | 0.14 | 0.3 | 0.23 | 0.11 | 0.13 | 0.13 |
| NA, G | 0.1 | 0 | 0.06 | 0.16 | 0 | 0 | 0.17 | 0 | 0 | 0.07 | 0.1 | 0 |
| Mogibacterium, G | 0.14 | 0 | 0.06 | 0.08 | 0 | 0 | 0.08 | 0 | 0 | 0.1 | 0 | 0 |
| **P:** Firmicutes ; **C**: Clostridia ; **O**: Clostridiales **: F:** Clostridiaceae_1 | | | | | | | | | | | | |
| Clostridium_sensu_stricto_1, **G** | 0.1 | 0 | 0.2 | 0.1 | 0 | 0 | 0.28 | 0 | 0 | 0 | 0.15 | 0.19 |
| **P:** Firmicutes ; **C:** Clostridia ; **O**: Clostridiales | | | | | | | | | | | | |
| Peptostreptococcaceae, **F**; Romboutsia, **G** | 0.07 | 0 | 0.06 | 0 | 0 | 0 | 0.08 | 0 | 0 | 0 | 0 | 0.06 |
| Peptococcaceae, **F**; NA, **G** | 0.14 | 0 | 0 | 0 | 0 | 0.1 | 0 | 0 | 0 | 0 | 0 | 0.09 |
| **P**: Firmicutes ; **C**: Erysipelotrichia ; **O**: Erysipelotrichales | | | | | | | | | | | | |
| Erysipelotrichaceae, **F** ; NA, **G** | 0.13 | 0 | 0.07 | 0 | 0 | 0 | 0 | 0.09 | 0 | 0 | 0 | 0 |
| **P**: Firmicutes ; **C**: Bacilli | | | | | | | | | | | | |
| Lactobacillales, **O**; Lactobacillaceae, **F**; Lactobacillus, **G** | 0.6 | 0.4 | 0.08 | 0 | 0 | 0 | 0 | 0 | 0 | 0 | 0.06 | 0 |
| Bacillales, **O**; Bacillaceae, **F**; NA, **G** |  |  | 0.1 |  |  |  |  |  |  |  |  |  |
| **P**: Planctomycetes ; **C**: Planctomycetacia;**O**: Pirellulales : **F**: Pirellulaceae | | | | | | | | | | | | |
| p-1088-a5_gut_group, **G** | 0.5 | 0.4 | 0.75 | 0.26 | 0.42 | 0.07 | 1 | 0.4 | 0.4 | 0.33 | 0.67 | 0.4 |
| CPla-4_termite_group**, G** | 0.21 | 0.42 | 0.25 | 0 | 0.2 | 0 | 0 | 0 | 0 | 0 | 0 | 0.09 |
| Pirellula, **G** | 0.1 | 0.08 | 0.07 | 0 | 0 | 0.02 | 0.06 | 0 | 0 | 0.06 | 0 | 0 |
| **P**: Proteobacteria ; **C**: Gammaproteobacteria; **O:** Aeromonadales: **F: Succinivibrionaceae** | | | | | | | | | | | | |
| NA, **G** | 0.15 | 0.34 | 0.06 | 0 | 0.4 | 0.1 | 0 | 0 | 0 | 0 | 0.1 | 0 |
| UCG-002, **G** | 0.5 | 0.19 | 0 | 0 | 0.65 | 0.64 | 0.16 | 0.77 | 0 | 0 | 0 | 0.16 |
| **P**: Proteobacteria ; **C**: Gammaproteobacteria | | | | | | | | | | | | |
| Oceanospirillales, **O;** Halomonadaceae, **F**; Halomonas, **G** | 1.3 | 0.9 |  |  |  |  |  |  |  |  |  |  |
| Alteromonadales, **O;** Idiomarinaceae, **F;** Aliidiomarina, **G** | 0.1 |  |  |  |  |  |  |  |  |  |  |  |
| **P**: Proteobacteria ; **C**: Deltaproteobacteria; **O**: Desulfovibrionales: **F**: Desulfovibrionaceae | | | | | | | | | | | | |
| Desulfovibrio, **G** | 0.17 | 0.19 | 0.1 | 0 | 0.21 | 0.1 | 0.13 | 0.17 | 0.16 | 0.11 | 0.17 | 0 |
| **P**: Spirochaetes ; **C:** Spirochaetia; **O**: Spirochaetales: **F**: Spirochaetaceae | | | | | | | | | | | | |
| Treponema_2, **G** | 1.2 | 1.03 | 0.7 | 1.2 | 0.6 | 0.86 | 0.75 | 0.26 | 0.4 | 1.3 | 0.67 | 0.8 |
| Sphaerochaeta, **G** | 0 | 0 | 0.06 | 0 | 0.15 | 0 | 0 | 0.16 | 0 | 0.13 | 0 | 0.15 |
| Sediminispirochaeta, **G** | 0.09 | 0.11 | 0.08 | 0.1 | 0.1 | 0.1 | 0.13 | 0.13 | 0 | 0.1 | 0.1 | 0.1 |
| **P:** Synergistetes ; **C**: Synergistia; **O**: Synergistales: **F**: Synergistaceae | | | | | | | | | | | | |
| Fretibacterium, **G** | 0.08 | 0.15 | 0.1 | 0.12 | 0.19 | 0.03 | 0.18 | 0.29 | 0.2 | 0.1 | 0.43 | 0 |
| Pyramidobacter, **G** | 0.07 | 0 | 0 | 0 | 0.13 | 0.1 | 0 | 0.1 | 0 | 0 | 0 | 0 |
| **P:** Tenericutes ; **C:** Mollicutes | | | | | | | | | | | | |
| Mollicutes_RF39, **O;** NA, **F;** NA, **G** | 1.9 | 1.19 | 1.8 | 1 | 0.2 | 0.35 | 1.6 | 1 | 0.29 | 1.06 | 0.73 | 0.57 |
| Anaeroplasmatales, **O;** Anaeroplasmataceae, **F;** Anaeroplasma, **G** | 0 | 0 | 0 | 0 | 0 | 0.07 | 0 | 0 | 0 | 0.11 | 0 | 0.2 |
| **P**: Verrucomicrobia ; **C:** Verrucomicrobiae; **O**: LD1-PB3: **F:** NA | | | | | | | | | | | | |
| NA, **G** | 0.16 | 0 | 0.02 | 0 | 0 | 0 | 0 | 0.07 | 0 | 0 | 0.08 | 0 |

P =Phylum, C= class, O=order, F= family, G=genera
