# Supplementary material for: Changes in the bacterial community colonizing extracted and non-extracted tannin-rich plants in the rumen of dromedary camels
Source: PLoS One. 2023 Mar 10;18(3):e0282889. doi: 10.1371/journal.pone.0282889 (PMC10004507; doi:10.1371/journal.pone.0282889)
Supplement: S2 Table — (DOCX) [file pone.0282889.s002.docx]

Supplementary Table S2: Comparison of the relative abundances (%) of the main rumen bacteria in camels of current study and previous study and other ruminant animals.

|  | Camel | | Sheep [58] | Cow [59] | Buffalo [59] |
| --- | --- | --- | --- | --- | --- |
|  | Current study | Camel [ 41]* |  |  |  |
| Prevotella | 31.56 | 4.15 | 17.4 | 53.6 | 26.5 |
| RC9_gut_group | 6.3-8 | 1.7 | 16.7 | 6.02 | 1.01 |
| Fibrobacteria | 0.03-0.1 | 4.4 | 0.18 | 0.01 | 0.29 |
| Ruminococcaceae | 3-7.3 | 15.66 | 5.9 | 1.51 | 18.96 |
| Butyrivibrio | 0.2-1 | 2.58 | 0.7 | 1.69 | 2.09 |
| Treponema | 0.4-1.3 | 3.85 | 0.8 | 0 | 0 |
| Selenomonas | 0.46-4.6 | 0.4 | 0 | 0 | 0 |
| Anaeroplasma | 0.07-0.11 | 0.62 | 0.15 | 0 | 0 |

*Animal fed Egyptian clover hay
