# Supplementary material for: Changes in the bacterial community colonizing extracted and non-extracted tannin-rich plants in the rumen of dromedary camels
Source: PLoS One. 2023 Mar 10;18(3):e0282889. doi: 10.1371/journal.pone.0282889 (PMC10004507; doi:10.1371/journal.pone.0282889)
Supplement: Attachment — Submitted filename: review- plos one.pdf [file pone.0282889.s004.pdf]

Comments

Line 19 add and semi-arid before countries.

Line 31 you may add one sentence about the impact of the study on improving the animal performance.

Line 41 add alternative before solutions.

Line 51 add plant before species.

Line 58 add process after fermentation.

Line 82 change on to in.

Line 101 change female camels to be she-camels through all the manuscript.

Line 111 why the author select only two incubation times at 6 and 12 h …..For example why he did not increased the incubation period to reach 24 h or 48 hours.is there an explanation for that.

Line 174 add a legend for the table containing the full name of the abbreviations CP, NDF and ADF.

Line 190 same as the previous comment.

P value in table 2, 3, 4 and 5 to be centered.

In the references part the doi name should be stable through all the references to be as follows https://doi.org/10.1007/s11250-011-9966-2.
