# Supplementary material for: Changes in the bacterial community colonizing extracted and non-extracted tannin-rich plants in the rumen of dromedary camels
Source: PLoS One. 2023 Mar 10;18(3):e0282889. doi: 10.1371/journal.pone.0282889 (PMC10004507; doi:10.1371/journal.pone.0282889)
Supplement: Attachment — Submitted filename: 000-Response to Reviewers.docx [file pone.0282889.s006.docx]

Desert Research Center	22^th^ January 2023

1Mathaf El Matariya St.B.O.P.11753

Matariya- Cairo,Egypt

Phone: (+202)26332846 - 26374800

FAX: (+202) 26357858

Email: [Rabee_a_m@yahoo.com](mailto:Rabee_a_m@yahoo.com)

PlOS ONE editorial office

**Changes in the bacterial community colonizing extracted and non-extracted tannin-rich plants in the rumen of dromedary camels**

We thank you and the Reviewers for their constructive comments and corrections. We responded to all comments and have extensively modified many sections in the paper to correct grammatical and style errors. I also enclosed unclean paper including all comments colored by yellow. Below are the responses to all the comments. Our paper was improved greatly after the response to the reviewers’ comments.

We appreciate the opportunity to submit our manuscript to PlOS ONE.

Yours sincerely,

Dr. Alaa Rabee

Researcher at Desert Research Center, Egypt

On behalf of all authors

**Comments**

Manuscript title: **“Changes in the bacterial community colonizing extracted and non-extracted tannin-rich plants in the rumen of dromedary camels”**

In the unclean or marked manuscript you could notice that colored comments using yellow.

**Responses**

**Reviewer 1**

**The primary concern in this study is the number of samples used to study the plant-attached bacterial community. The authors (lines 109–118) used only one bag for each category of sample (plant type, sample, incubation time) per animal. For each sample, one bag per animal is insufficient to capture the bacterial community.**

>> Thank you for your comment, most of the studies use one bag per every type of sample to study the microbial community attached to the plants, and this is completely correct, especially we used three animals per group. However, using more than one bag is better, especially if the authors have enough funds. In our case, we used one bag and only two-time points due to the lack of fund.

**The authors did not run a permutational multivariate ANOVA (PERMANOVA) or other analysis to see if there were any statistical differences between bacterial communities for beta diversity analysis (lines 198–201).**

>> Thank you for your advice, we conducted PERMANOVA and modified the material and results sections.

**Line 133-134: Several studies have shown that higher PCR cycle numbers result in chimaera generation and interfere with the analysis of bacterial community structure (1 & 2).** **This study used 35 PCR cycles to amplify the targeted region that has a higher chance of nonspecific amplification and errors. Generally, researchers are using up to 25 PCR cycles to amplify the targeted region in rumen bacterial communities.**

>> Thank you so much for your comments, some Authors used these primers set for PCR amplification using 25 cycles and other used 35 cycles. However, an optimization study by Walters et al. (2015) used 35 cycles. Therefore, we used 35 cycle in our study.

**Lines 76–78: Another aim of the study was to see the degradation of plant material, and this study only used two-time points (6 and 12 hours). These time points are not enough to see the degradation of the plant materials.**

>> Thank you, we wanted to link the degradation with the changes in microbial communities, but due to the lack of the funds we used only two times. However, in the future work we will avoid this point.

**Reviewer 2**

**Line 19: add and semi-arid before countries.**

>>Thank you for your comments, modified

**Line 31: you may add one sentence about the impact of the study on improving the animal performance.**

>> added

**Line 41: add alternative before solutions.**

>> added

**Line 51: add plant before species.**

>> added

**Line 58: add process after fermentation.**

>> added

**Line 82: change on to in.**

>> Changed

**Line 101: change female camels to be she-camels through all the manuscript.**

>> Changed

**Line 111: why the author select only two incubation times at 6 and 12 h …..For example why he did not increased the incubation period to reach 24 h or 48 hours.is there an explanation for that.**

>> Thank you for your comment; we wanted to link the plant degradation with the changes in bacterial communities attached the plants. However, due to the lack of the fund we used only two incubation times. I hope we avoid this point in the future studies.

**Line 174: add a legend for the table containing the full name of the abbreviations CP, NDF and ADF.**

>> Thank you, we added it.

**Line 190: same as the previous comment.**

>> Thank you, we added it.

**P value in table 2, 3, 4 and 5 to be centered.**

>> Fixed

**In the references part the doi name should be stable through all the references to be as follows** [**https://doi.org/10.1007/s11250-011-9966-2**](https://doi.org/10.1007/s11250-011-9966-2)**.**

>>Modified

**Reviewer 3**

**-Please add the P value in the results of the abstract and the results part.**

>> Thank you for your comments, added.

**-Please check that all abbreviations were provided in full names in their first mention and do not repeat.**

>> Checked and modified, thank you.

**- Line 50-51: In response to this sentence, the authors can say camel can utilize poor-quality fodder plants. Please authors can take a deeper look at this recent study it may be useful for the introduction and the discussion part as well.**

**Khattab, I.M., Abdel-Wahed, A.M., Anele, U.Y., Sallam, S.M. and El-Zaiat, H.M., 2021. Comparative digestibility and rumen fermentation of camels and sheep fed different forage sources. Animal Biotechnology, pp.1-10. doi: 10.1080/10495398.2021.1990939.**

>> Modified, and we used the reference.

**-Please, insert the method to calculate DM, CP and NDF disappearance.**

>> Clarified, thank you.

**-Table 1: please, check the chemical composition of the experimental fodder shrubs CP, total phenols, and total tannins. The CP is very high. Atriplex has low total phenols and condensed tannin content at 0.2g/kg DM according to ref 4 and khattab, 2007 (Khattab IM. Studies on halophytic forages as sheep fodder under arid and semi arid conditions in Egypt. Ph. D. Thesis. Alexandria University; 2007.(10.13140/RG.2.2.20956.21128)). Please authors can take a deeper look at this PhD study that was carried out in the same place as the study.**

>> Thank you for this comment, we recalculated the chemical composition based on the Reviewer advice, but we got the same results. The difference in the chemical composition in our study and khattab, (2007) might be attributed to using different plant part or collecting the plants in different season, we modified the discussion based on that and referred to the reference.

**-Discussion needs to revise according to the chemical composition of fodder shrubs.**

>> Thank you, please see the response above.

**- Authors gave reasons for the low disappearance in acacia but did not give reasons for the high disappearance of Atriplex. The authors can benefit from this study, which shows the higher disappearance in the Atriplex due to the higher NPN content of the plant.
Khattab, I.M. and Anele, U.Y., 2022. Dry matter intake, digestibility, nitrogen utilization and fermentation characteristics of sheep fed Atriplex hay‐based diet supplemented with discarded dates as a replacement for barley grain. Journal of Animal Physiology and Animal Nutrition, 106(2), pp.229-238. https://doi.org/10.1111/jpn.13577**

>> Thank you for this comments, we modified the discussion accordingly, and we used this reference.

**Reviewer 4**

**The manuscript entitled “Changes in the bacterial community colonizing extracted and non-extracted tannin-rich plants in the rumen of dromedary camels.” by Alaa Emara Rabee et al. is well written manuscript and will be of interest to the science community in the field. I have few minor comments for correction in the manuscript attached and will recommend the manuscript for publication in Plos One after the minor comments were answered properly.**

Thank you for your comments; we answered your comments as indicated in the attached file.
